# *N*-Glycosylation Modification of Plant-Derived Virus-Like Particles: An Application in Vaccines

**DOI:** 10.1155/2014/249519

**Published:** 2014-05-25

**Authors:** Hyun-Soon Kim, Jae-Heung Jeon, Kyung Jin Lee, Kisung Ko

**Affiliations:** ^1^Plant Systems Engineering Research Center, KRIBB, 125 Gwahangno, Yuseong-gu, Daejeon 305-806, Republic of Korea; ^2^Department of Medicine, College of Medicine, Chung-Ang University, Seoul 156-756, Republic of Korea

## Abstract

Plants have been developed as an alternative system to mammalian cells for production of recombinant prophylactic or therapeutic proteins for human and animal use. Effective plant expression systems for recombinant proteins have been established with the optimal combination of gene expression regulatory elements and control of posttranslational processing of recombinant glycoproteins. In plant, virus-like particles (VLPs), viral “empty shells” which maintain the same structural characteristics of virions but are genome-free, are considered extremely promising as vaccine platforms and therapeutic delivery systems. Unlike microbial fermentation, plants are capable of carrying out *N*-glycosylation as a posttranslational modification of glycoproteins. Recent advances in the glycoengineering in plant allow human-like glycomodification and optimization of desired glycan structures for enhancing safety and functionality of recombinant pharmaceutical glycoproteins. In this review, the current plant-derived VLP approaches are focused, and *N*-glycosylation and its in planta modifications are discussed.

## 1. Plant-Derived Virus-Like Particle (VLP)

Viruses are able to form the quaternary structure of viral capsids through molecular self-assembly of repetitive building blocks [1, 2]. Plant viruses can be easily multiplied, which are structurally uniform, robust, and biodegradable with a size particularly suitable for nanoscale applications. Virus-like particles (VLPs) are multimeric self-assembled protein complexes mimicking the organization and conformation of native viruses but lack the viral genome making them replication-deficient and noninfectious [3]. VLPs consist of protein shells (termed as capsids), and the capsids are typically composed of identical coat protein subunits. Peptide-based vaccines are in general poorly immunogenic and for this reason they require multiple injections and adjuvants in order to increase their effectiveness. VLPs offer a promising approach to the production of vaccines against many diseases, because their repetitive, high density display of epitopes is potentially highly effective in eliciting strong immune responses [4]. VLPs lacking viral nucleic acid are noninfectious. Nevertheless, they are self-assembled protein structures mimicking infectious viruses and thus constitute a safe and effective approach for the induction of neutralizing antibodies to surface proteins, where soluble forms of their protein subunits have failed. It has been also reported that viral structures are regarded as a vaccine platform to display foreign epitopes [5].

In general, bacteria, yeast, insect, and animal cells have been applied as cell-based systems to produce VLPs. The bacterial cell cultures have been explored as a VLP production platform with advantages in terms of scalability and production cost [6]. However, bacteria are prokaryotes which lack glycosylation process essential for proper immunogenicity and antigen stability when VLPs are applied as vaccines. In contrast to bacteria, yeast cells have glycosylation apparatus [7]. However, their glycoforms are mainly high mannose type, which is not desirable for the most therapeutic glycoproteins [8]. The matured glycoforms in baculovirus-insect cell system also are mainly high mannose type [9]. The glycosylation of envelope proteins affects their folding and thus is essential for formation and immunogenicity of VLPs [10, 11]. In glycosylation process, bacteria, yeast, and insect cells have fundamental limitations. The mammalian cells have proper glycosylation apparatus and ability to fold the envelope proteins of virus, which facilitate functional VLPs production. However, the mammalian cell-based systems require manufacturing facilities including fermentation bioreactors for large-scale upstream processing, which is too expensive to establish. This high production cost is a major disadvantage of the mammalian cell-based system. Plants do not need such expensive facilities to produce biomass. Thus, plants are considered as a potential bioreactor system for VLPs with advantages such as low cost of upstream biomass process, flexible production scalability, and the lack of human pathogen contaminants [12, 13]. Nevertheless, plants for VLP production platform are not perfectly acceptable due to relatively lower VLP production level than animal systems and plant-specific* N*-glycosylation of glycoproteins [14, 15]. Development of new plant expression system and advanced* N*-glycosylation engineering overcome such hurdles.

## 2. Virus-Like Particles in Plant Expression Systems

VLPs can be generated through different types of viral vectors and expression strategies in plants [16, 17]. The plant-derived viral vectors used for VLP expression can be classified into full virus vectors such as the potato virus X (PVX) [18, 19] and the cowpea mosaic virus (CPMV) [20] and the deconstructed vectors such as bean yellow dwarf virus (BeYDV) [21, 22] and MagniCON based on tobacco mosaic virus (TMV) [23, 24]. The earlier plant VLPs were Hepatitis B core antigen (HBcAg) VLPs [25] and Hepatitis B surface antigen (HBsAg) VLPs fused to soybean vegetable storage protein vspA (VSP*α*S) in transgenic tobacco leaves obtained by* Agrobacterium*-mediated transformation [25, 26]. PVX and CPMV based viral vectors were applied to generate HBcAg VLPs [27]. Transgenic plants using* Agrobacterium*-mediated DNA transfer have been used for the stable gene expression system for VLPs; however the VLP expression level is low [10~24 *μ*g/g fresh leaf weight (FLW)] [27, 28]. The human papilloma virus (HPV) L-1 based VLP vaccines were also successfully expressed and assembled in stable transgenic potato and tobacco [29–31]. Unlike stable transformation, the transient expression with deconstructed geminiviral vectors showed 80 times higher accumulation of HBcAg VLPs in* N. benthamiana* compared to both PVX and CPMV vectors [32]. Another transient expression system using deconstructed TMV-based MagnICON vector agroinfiltration has allowed the production of HBcAg VLPs with a yield of 2.38 mg/g (FLW), almost 3 times higher compared to the geminiviral vector within a short period time [17, 32–35]. The intraperitoneally injected HBcAg VLPs obtained from the MagnICON system efficiently induced immune responses generating HBcAg specific IgGs in mice. These results indicate that, among currently available VLP expression systems, the production of large quantities of VLPs for vaccine applications is more feasible using MagniCON systems. In many cases genetic manipulation of plant-derived VLPs has been performed to modify the external surface of the particle. To this end, the heterologous polypeptide has been fused at the N- or C-terminus of the CP. VLPs can also be exploited as “platforms” for the presentation of foreign epitopes and/or targeting molecules on chimeric VLPs (cVLPs) [1, 2, 6]. Indeed, the VLPs can display multicomponent vaccine candidate epitopes as a fusion form between two different proteins [5]. For instance, the green fluorescent protein (GFP) and the HB surface antigen (HBsAg) S-protein were transiently expressed and heterodimerized with the native HBsAg sequentially forming chimeric VLPs (cVLPs) in* N. benthamiana* [36]. The HBsAg fusion with GFP was showed to be more stable and immunogenic than native HBsAg in* in vivo* mice experiment, indicating that cVLPs can be applied to display heterologous antigens to generate more immunogenic vaccines [5]. The fusion proteins between domain III (DIII) of West Nile virus (WNV) and HBcAg were expressed and displayed as cVLPs with geminiviral transient expression vectors in* N. benthamiana* [37]. In addition, the influenza virus M2 epitope [38] or HPV16 epitopes [27, 39, 40] individually were fused to HBcAg induced strong immune responses generating specific antibodies. The cVLPs displaying both HPV16 E6 and E7 proteins triggered their specific antibodies, respectively [39]. In general, vaccines are administered through intramuscular, subcutaneous, and intravenous injections. In addition, vaccines can be orally or nasally applied to induce mucosal immune responses [17]. Indeed, various results indicate that VLPs can be applied safely as oral vaccines carrying multiple epitopes without needle injection. For example, oral delivery of purified Norwalk virus CP (NVCP) VLPs produced in tobacco and tomato stimulated mucosal and serum immune responses to produce IgA and IgG [41] and oral administration with HBsAg displaying HIV-1 ENV and GAG epitopes provoked strong serum and mucosal antibody responses in mice [42]. These results indicate that VLPs can be applied safely as oral vaccines carrying multiple epitopes without needle injection.

## 3. Glycosylation of VLP Vaccines

Even though virus-like particles- (VLPs-) based vaccines have shown promising results, commercial production systems are currently limited to eukaryotic cells such as yeast, insect, and mammalian [14]. For instance, Lassa virus (LASV) VLPs cannot be easily produced in bacterial cell systems, because bacteria are incapable of performing glycosylation and other posttranslational protein modifications which are a key feature in most VLP-based proteins [14]. The glycosylation pattern of GP1 and GP2 glycoproteins of Lassa virus (LASV) has been shown to play a critical structural and functional role in preserving protein stability and allowing binding and fusion to host cells [43]. The glycosylation of VLP proteins has major impact on their structure and function, and thus it is important to determine the choice of platforms for their production. As the viral glycoproteins localize, guide, and potentiate the process of enveloped virus assembly, it becomes important to study their individual and combined behavior upon expression in both animal and plant cells, in order to identify domains within the glycoproteins responsible for the critical differences between the intracellular targeting in either cell system. The large structural protein of lettuce necrotic yellow virus was glycosylated with complex oligosaccharides containing* N*-acetylglucosamine* N*-linked to asparagine residues [44]. The potato virus X CP and PPV CP were also glycosylated [45]. Glycosylated CP of beet western yellows virus plays a role in the virus/aphis interaction and promotes the aphid transmission of the virus [46]. Reviewed earlier, plants offer an attractive alternative system for VLP vaccine production with cost-effective, scalable, versatile, appropriate glycosylation, efficient assembly of VLP, and safety from adventitious human pathogens [12].

**Figure 1:**
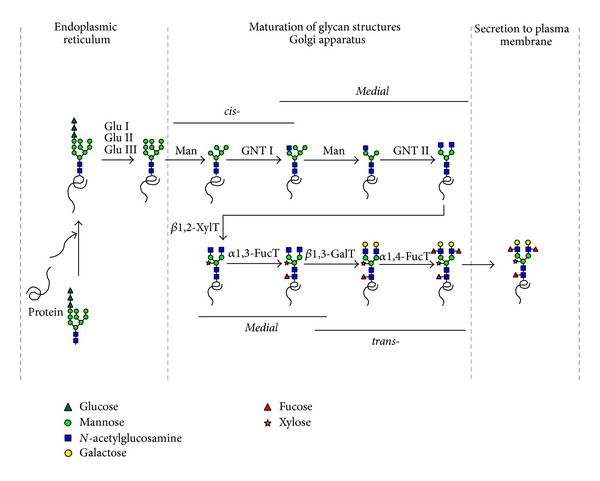
*N*-glycosylation pathway in plant. The primary glycosylation pathways with consequent series of steps occur in different subcellular compartments, ER, Golgi complex within the plant cell. During the pathway, glycosidase digestion and additional glycosyltransferase result in additional different branches and terminal glycan residues. GuI: glucosidase I, GuII: glucosidase II, GuIII: glucosidase III, Man: mannosidase, GNT I:* N*-acetylglucosaminyltransferase I, GNT II:* N*-acetylglucosaminyltransferase II, *β*1,2-XylT: *β*1,2-xylose transferase, *α*1,3-FucT: *α*1,3-fucose transferase, *β*1,3-GalT: *β*1,3-galactosidase, *α*1,4-FucT: *α*1,4-fucose transferase.

**Figure 2:**
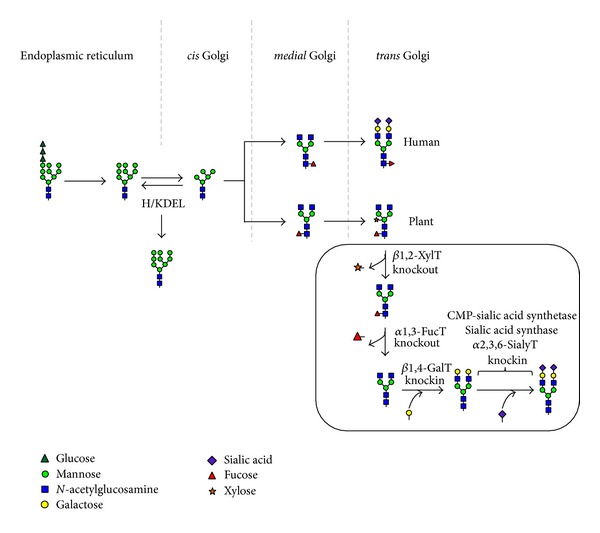
Schematic diagram of humanization of the glycosylation pathway in plant. In ER, protein is glycosylated and three glucoses are removed from the attached glycan. The glycoproteins then are transferred to the Golgi complex where mannoses are trimmed, and the glycoresidues are sequentially attached. When the ER retention signal KDEL sequence is attached to the C-terminal of glycoproteins, glycoproteins are retained and accumulated in the ER. Plant glycans carry *β*1,2-xylose and *α*1,3-fucose residues attached to the* N*-acetylglucosamine whereas human glycans contain *α*1,6-fucose, *β*1,4-galactose, and *α*2,3,6-sialic acid. In humanization glycoengineering process the *β*1,2-XylT and *α*1,3-FucT should be knocked out to remove xylose and fucose, respectively. The *β*1,4-GalT should be knocked in to add *β*1,4-galactose. Furthermore, finally CMP-sialic acid synthetase, sialic acid synthase, and *α*2,3,6-sialic transferase should be knocked in to attach *α*2,3,6-sialic acid to the terminal galactose. KDEL: ER retention motif (Lys-Asp-Glu-Leu), *β*1,2-XylT: *β*1,2-xylose transferase, *α*1,3-FucT: *α*1,3-fucose transferase, *β*1,4-GalT: *β*1,4-galactosidase, *α*2,3,6-SialyT: *α*2,3,6-sialic transferase.

Although it yielded encouraging results, expression of VLPs expressed in plants suffers from plant-specific glycosylation of glycoproteins [14, 15]. Most proteins in eukaryotic multicellular organisms including plants are synthesized as glycoproteins with* N*- and* O*-glycosylation, which are important posttranslational protein modifications [47].* N*-glycans attached to proteins are crucial for protein folding, assembly, and their stability but also involved in cell to cell adhesion, protein targeting, and immune responses as biological activity [48, 49]. During the glycoprotein transportation through the secretory pathway, the oligosaccharide* N*-linked to the asparagine residue (Asn) undergoes several maturation steps involving the removal of glucose and mannose residues by different exoglycosidase to generate high mannose type* N*-glycan in the endoplasmic reticulum (ER) and the Golgi apparatus and, eventually, it is characterized by the addition of new oligosaccharide residues in the Golgi apparatus to form the matured complex type* N*-glycan (Figure 1). In the ER, the first step of* N*-glycosylation of plant proteins is the transfer of the oligosaccharide precursor Glc_3_Man_9_GlcNAc_2_ from a dolichol lipid to specific Asn residues on the nascent polypeptide chain [50]. Processing of this oligosaccharide into high mannose, complex, hybrid, or paucimannosidic type* N*-glycan occurs during the secretory pathway. Particularly in plant, the *β*-mannose is substituted by a bisecting *β*1,2-xylose that is not found in mammalian* N*-glycans, and the proximal* N*-acetylglucosamine of the core is substituted by an *α*1,3-fucose, instead of an *α*1,6-fucose in mammalians. In addition, *β*1,3-galactose and fucose that are *α*1,4-linked to the terminal* N*-acetylglucosamine of plant* N*-glycans form Lewis a (Le^a^) glycosylation (Figure 1) [51]. These modifications are not present in mammalian. Many mammalian complex* N*-glycans have an *α*1,6-fucose on the first core* N*-acetylglucosamine of* N*-glycan and are characterized by terminal *β*1,4-galactose and sialic acid which are not observed in plants (Figure 1) [52–54]. Most plant-derived therapeutic proteins are complex glycoproteins requiring posttranslational modifications. The *β*1,2-xylose, core *α*1,3-fucose, and Le^a^ containing epitopes have been considered as immunogenic glycan epitopes found in plant-specific* N*-glycans. Such glycan residues are not present in humans, and thus proteins could cause immune rejection inducing plant-glycan specific antibodies causing protein clearance in blood stream as well as potential allergenic effects [54–56]. These hurdles can be overcome by recent progress in plant glycoengineering. The plant expression with glycoengineering will allow the novel application of plant-made VLPs, including vessels for the delivery of small therapeutics, DNA fragments, and adjuvants (Figure 2).

## 4. *N*-Glycomodification in Plants

### 4.1. Targeted Expression to the ER

*N*-glycan structures influence biofunctionality and stability of therapeutic proteins and even directly affect immunogenicity of glycosylated subunit vaccines displayed on VLP surfaces. In plants, thus,* N*-glycosylation pathway has been modified in order to humanize the glycan structures of glycoproteins [57, 58].

A commonly used approach to express recombinant glycoproteins in plants is their accumulation in ER by addition of C-terminal signal H/KDEL ER retention motif [59]. The ER-retained proteins contain high mannose type* N*-glycans structurally similar between plant and mammalian cells [58, 60, 61]. The high mannose type* N*-glycans are oligosaccharide structures that mammals and plants have in common and thus are probably not immunogenic [62]. This strategy is largely devoid of plant-specific, immunogenic *β*1,2-xylose and core *α*1,3-fucose. Additionally, some studies have reported enhanced accumulation of KDEL-tagged proteins in the ER. Such ER retention of proteins usually increases the production level compared to that without KDEL in plant [63, 64]. Plant-derived monoclonal antibody (mAb) with high mannose* N*-glycan structure has shorter half-life than that of the mammalian-derived mAb with mammalian specific glycan structures [65]. However, mAb with high mannose glycans had relatively similar biological activities compared to the mammalian-derived mAb overcoming concerns about plant-specific glycoepitopes expressed by others [58]. In addition, the high mannose type glycan structure would be expected to cause an enhanced immune response through the mannose receptor (MR) on macrophages and dendritic cells recognizing the oligomannose of glycoproteins [66], which is an advantage for vaccine development. According to a previous study [67], the high mannose glycans on antigenic protein can render the protein more immunogenic, producing IgG against the high mannose glycosylated protein.

### 4.2. Knockout of Plant-Specific Glycosyltransferases

Gene inactivation or silencing may be used to reduce or eliminate the activity of plant- specific glycosyltransferases. In a plant cell, the specific enzymes are *β*1,2-xylosyltransferase and core *α*1,3-fucosyltransferase, which are responsible for transfer of the plant-specific xylose and fucose onto the attached* N*-glycan. Such glycan residues are not present in humans and are thus unwanted on proteins intended for therapeutic use. The knockout of the genes that are responsible for the synthesis of these glycan epitopes *β*1,2-xylosyltransferase and core *α*1,3-fucosyltransferase provides an easy strategy to solve this problem. The feasibility of this strategy has been proven by the generation of knockout* Arabidopsis thaliana* plant lacking xylosyltransferase and fucosyltransferase [68, 69]. In addition, biological activity assays of such glycoengineered mAbs showed that their antigen binding activity was not altered but significantly enhanced antibody-dependent cell-mediated cytotoxicity (ADCC) effect [70, 71]. Therapeutic antibodies without fucosylation have higher binding affinity for Fc*γ*RIIIa than for fucosylated human serum IgG, which is desirable to overcome the interference by human plasma IgG. Thus, the therapeutic antibodies without fucosylation can avoid the inhibitory effect of human plasma IgG on ADCC through their high Fc*γ*RIIIa binding affinity.

### 4.3. Humanization of Plant* N*-Glycosylation

The immunogenic and allergenic reactions of the *β*1,2-xylose and core *α*1,3-fucose* N*-glycan epitopes on plant-derived glycoproteins have been a problem for application of therapeutic proteins produced from plant expression system [72]. Glycoengineering strategies using transgenic plants and the availability of mutant plants lacking xylosyltransferase and fucosyltransferase genes for humanization of* N*-glycosylation allow producing recombinant proteins with more mammalian-like* N*-glycan structures in plant expression system. Most proteins used for therapy of human diseases are glycosylated, and the glycan structures have been shown to affect safety and efficacy of therapeutic glycoproteins [73]. Particularly, nonsialylation significantly causes shorter* in vivo* half-life of circulating glycoproteins, because exposed galactose glycan residues are recognized and captured by asialoglycoprotein receptors resulting in internalization of the glycoproteins in hepatocytes [74]. Terminal acid residues in* N*-linked glycans of most therapeutic glycoproteins affect important roles in* in vivo* physical stability, immunogenicity, and enzymatic activity [68, 70, 71, 74, 75]. Previous studies have demonstrated the importance of fully sialylated* N*-linked glycans and of consistency of homogeneous* N*-linked glycan structures on therapeutic glycoproteins in heterologous expression systems [75–77]. For instance, the sialylated recombinant erythropoietin (EPO) had longer plasma half-life (5-6 h) compared to that (2 min) of desialylated EPO [78]. This beneficial effect of sialic acid on protein stability likely explains why knockin strategies for plant glycoengineering in glycosylation have mainly focused on the addition of terminal *β*1,4-galactose and sialic acid residues to humanize* N*-glycan in mutant plants lacking plant-specific* N*-glycan residues [75]. It was claimed that plant virus-based transient expression systems can be applied as the knockin strategy of *β*1,4-galactose and sialic acid transferring genes in the mutant plants, allowing the generation of abundant amount of therapeutic proteins within 1 week after virus infection, provide a feasible advantage over existing glycoprotein expression systems [75].

## 5. Conclusions

Taken together, plant-derived VLPs are considered safe because plants do not bear human pathogens and promising in terms of cost-effective scalability and speed of production. In fact, as far as upstream and downstream processing are concerned, plant-derived VLPs can take advantage of what has been done so far in the broader field of plant-made pharmaceuticals. Also compared to prokaryotes host cells, plants host guarantees the appropriate posttranslational modifications, such as glycosylation, often needed for proper protein function.

In plants, glycoengineering has been improved to create plants able to perform the ideal glycosylation enhancing efficacy and potency of VLPs-based therapeutics. As described in this review, several strategies, focused on the inactivation and/or addition of key enzymes, can be adopted to decorate tailor-made glycoforms of VLPs in plants. Thus, plant expression systems will be further improved for production of VLPs-based vaccines with respect to their proper glycomodification and the rapid and cost-effective expression.
